# Comparative profiling of well-defined copper reagents and precursors for the trifluoromethylation of aryl iodides

**DOI:** 10.3762/bjoc.13.225

**Published:** 2017-10-30

**Authors:** Peter T Kaplan, Jessica A Lloyd, Mason T Chin, David A Vicic

**Affiliations:** 1 Department of Chemistry, Lehigh University, 6 E. Packer Ave., Bethlehem, PA 18015, USA

**Keywords:** benchmarking, copper, fluorine, fluoroalkylation, trifluoromethylation

## Abstract

A number of copper reagents were compared for their effectiveness in trifluoromethylating 4-iodobiphenyl, 4-iodotoluene, and 2-iodotoluene. Yields over time were plotted in order to refine our understanding of each reagent performance, identify any bottlenecks, and provide more insight into the rates of the reactions. Interestingly, differences in reactivity were observed when a well-defined [LCuCF_3_] complex was employed directly or generated in situ from precursors by published reports. Relative reactivities were also found to highly dependent on the nature of the iodoarenes.

## Introduction

**Scheme 1:**
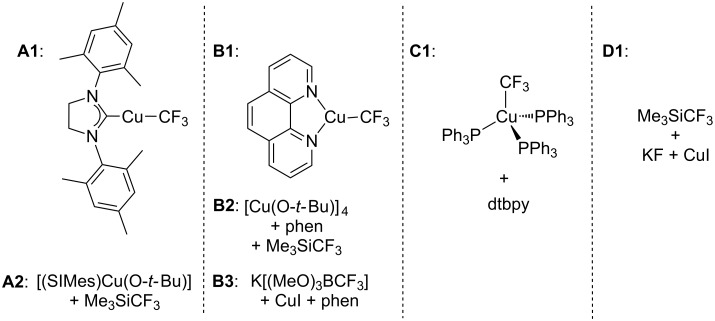
Reagents and precursors used for trifluoromethylation reactions.

**Scheme 2:**
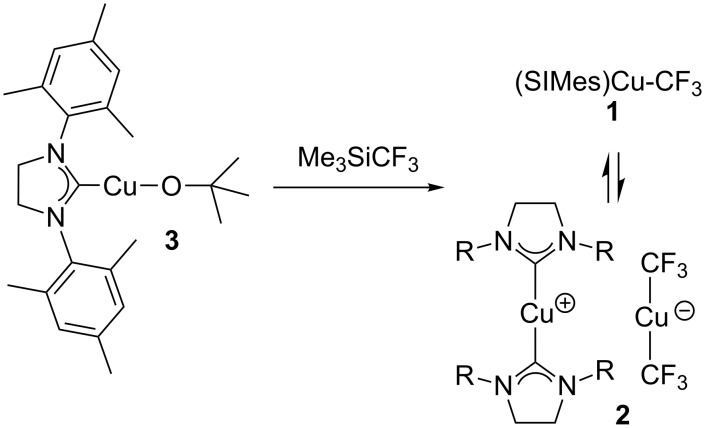
Preparation of [(SIMes)_2_Cu][Cu(CF_3_)_2_].

Selectively fluorinated molecules that bear the trifluoromethyl group have great importance in the life sciences and materials fields as well as discovery chemistry in general [1–3]. Consequently, transition-metal-catalyzed methods for preparing aromatic trifluoromethyl compounds from readily available aryl halides are an area that has seen rapid growth in the past ten years. Copper is one of the most successfully used metals for mediating the trifluoromethylation of aryl halides, and the active form of the reagents is typically a copper(I) complex bearing a trifluoromethyl ligand, i.e., [L*_n_*Cu–CF_3_]. Sporadic examples of trifluoromethylation ‘catalysis’ using copper have been observed [4–9], but these reactions typically only work for aryl iodides and have a low substrate scope, low turn-over values, and/or involve decarboxylation reactions at high temperatures. Stoichiometric trifluoromethylating agents are therefore more commonly used in benchtop trifluoromethylation chemistry. Ancillary ligands (L) are known to play a large role in the reactivity of such [L*_n_*Cu–CF_3_] reagents, and recent work has focused on developing new ligands that not only allow for better control of reactivity but also provide stability to facilitate meaningful comparative studies, such as structural and electrochemical ones [2]. *N*-Heterocyclic carbene (NHC) complexes of copper such as **A1** (Scheme 1) were the first well-defined and structurally characterized copper–CF_3_ complexes that display activity for the trifluoromethylation of aryl halides [10–11]. [(SIMes)CuCF_3_] (**1**, SIMes = 1,3-bis(2,4,6-trimethylphenyl)-4,5-dihydroimidazol-2-ylidene), which is in equilibrium with [(SIMes)_2_Cu][Cu(CF_3_)_2_] (**2**), can either be used directly or prepared in situ through the reaction of [(SIMes)Cu(O*-t-*Bu)] (**3**) with Me_3_SiCF_3_ (Scheme 2) [10]. Phenanthroline complexes of copper **B1** were reported shortly after the NHC counterparts [5,12] and have reached much success in chemical synthesis due to the ease of preparation and the low cost of the phenanthroline ancillary ligand. [(phen)CuCF_3_] can now be purchased commercially, or prepared in situ by a variety of methods including the reaction of [Cu(O*-t-*Bu)]_4_ with Me_3_SiCF_3_ and phen (**B2**, Scheme 1) [12] or by reaction of [(MeO)_3_BCF_3_] with CuI and phen (**B3**, Scheme 1) [8]. The compound [(PPh_3_)_3_CuCF_3_] has been for a long time [13], however, its trifluoromethylating ability and structure determination was not reported until 2011. Trifluoromethylations with [(PPh_3_)_3_CuCF_3_] are only efficient when the reactions are performed in neat aryl iodode [14]. Less side-products and higher yields are observed for trifluoromethylations with [(PPh_3_)_3_CuCF_3_] when dtbpy (dtbpy = 4,4′-di-*tert*-butylbipyridine) is added to reaction mixtures (**C1**) to presumably generate a dtbpy complex of CuCF_3_ [14]. Finally, conditions that generate “ligandless” [CuCF_3_] (**D1**, for example) are also amenable for the trifluoromethylation of aryl iodides [15], but it is unclear how the reactivity profile of the ligandless complex compares to systems **A**–**C** described in Scheme 1. An important issue is that only single time point yields have been reported for systems **B**–**D**, and the significantly different reaction conditions employed for each system have made it impossible to truly compare reagent performance based on the available literature data. For this reason, we sought to run trifluoromethylation reactions with systems **A**–**D** under both identical and the reported optimal reaction conditions in order to track how yields change over time for each reagent for comparative studies. We also sought to explore whether there were differences in reactivity when a well-defined [LCuCF_3_] complex was employed directly or generated in situ by published reports. If so, it will be informative to know the extent of differences in reagent performance over time.

## Results and Discussion

**Scheme 3:**
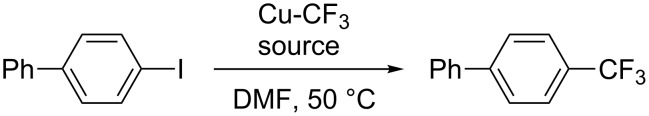
General protocol for reactions described in Figure 1.

**Figure 1:**
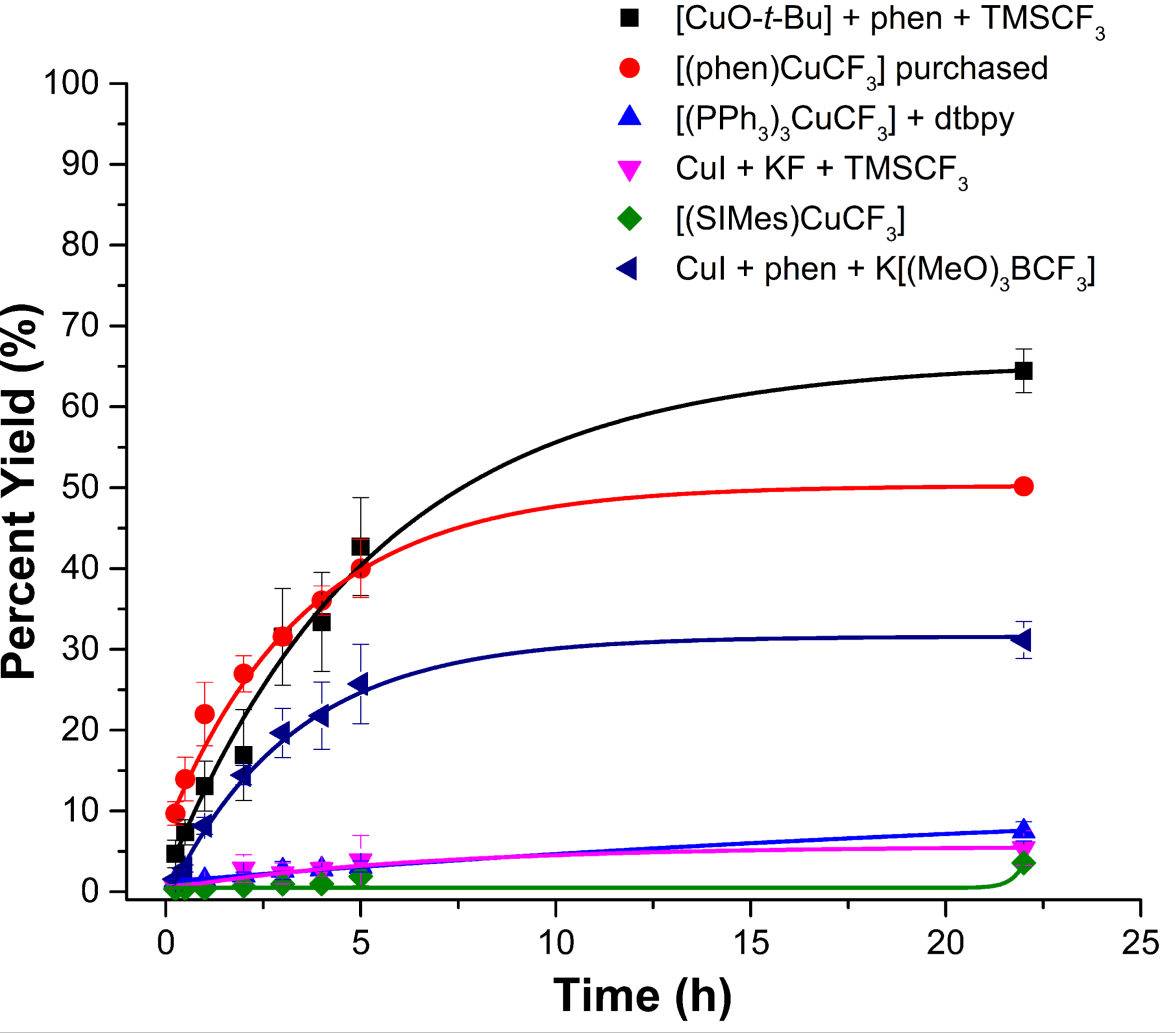
Yields of 4-(trifluoromethyl)-1,1’-biphenyl over time for the systems described in Scheme 1. These runs represent “standard” conditions described in Scheme 3 and the Experimental section. Yields were monitored by gas chromatography relative to a calibrated internal standard.

Because the phenanthroline-based system described as **B1** (Scheme 1) is the most widely used reagent for trifluoromethylations, we modeled our “standard” comparative conditions similar to those reported by Hartwig in 2011 [12]. These conditions involve reacting 4-iodo-1,1’-biphenyl with a [Cu–CF_3_] source at 50 °C in DMF (Scheme 3). Somewhat more diluted reaction conditions relative to the published procedure were used to ensure homogeneity for all the different complexes described in Scheme 1. Conversions to 4-(trifluoromethyl)-1,1’-biphenyl were then monitored by gas chromatography relative to a calibrated internal standard. Experiments were performed in triplicate, and the average yields over time are plotted graphically in Figure 1.

As shown in Figure 1, conditions where the [(phen)CuCF_3_] was generated in situ (**B2**) provided the best yields of 4-(trifluoromethyl)-1,1’-biphenyl after 24 hours, with yields and conversion of aryl iodide (data not shown) both near 65%. When commercially purchased [(phen)CuCF_3_] (**B1**) was used, yields up to the five hour mark were comparable to those of **B2**, but were ≈15% lower after the full 24 hours. Yields of product were 50%, with consumption of biphenyl iodide at 62%. Importantly, traditional single time point yields would not be able to highlight the loss of activity at the longer reaction times. The related phen system **B3**, which uses K[(MeO)_3_BCF_3_] as the trifluoromethyl source, performed significantly lower than **B1** and **B2** and gave a final overall yield of 31%. Consumption of the biphenyl iodide was found to be 51%. The data is intriguing because systems **B1**, **B2**, and **B3** are all expected to involve [(phen)CuCF_3_] as the active trifluoromethylating agent, yet there are clear differences in reactivities for only slight changes in chemical components in the reaction mixtures. For the same conditions in DMF at 50 °C, **A1**, **C1**, and **D1** all performed poorly relative to [(phen)CuCF_3_] and gave product yields less than 10% (Figure 1).

**Figure 2:**
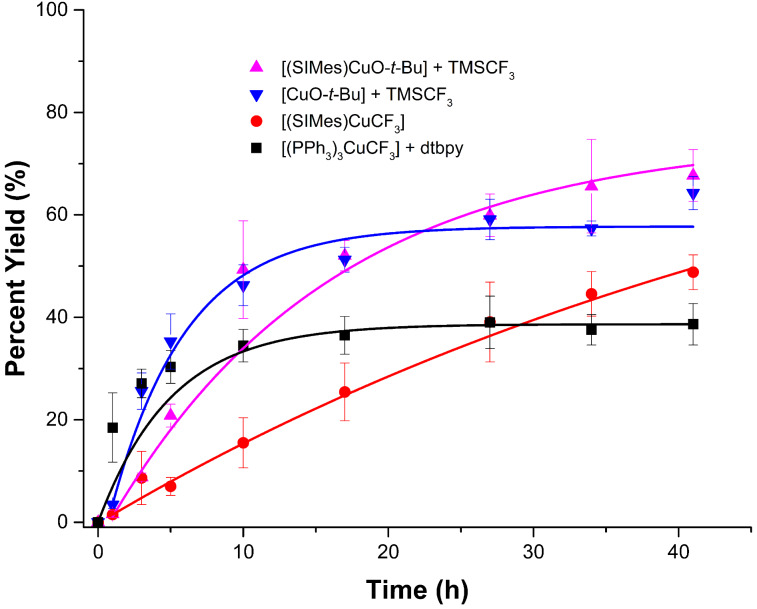
Yields of 4-(trifluoromethyl)-1,1’-biphenyl over time for the systems described in Scheme 1. Conditions for **C1**: [(PPh_3_)_3_Cu(CF_3_)] + dtbpy: toluene, 80 °C. Conditions for **A1**: [(SIMes)Cu(CF_3_)] and **A2** ([(SIMes)Cu(O-*t*-Bu)] + TMSCF_3_: DMI/benzene (1.5:7.5), 50 °C. DMI = 1,3-dimethyl-2-imidazolidinone. Conditions for **B2**: [(Cu(O-*t*-Bu)]_4_ + phen +TMSCF_3_: DMF, 50 °C. Yields were monitored by gas chromatography relative to a calibrated internal standard.

We then compared **B2**, the highest performing [(phen)CuCF_3_] system in DMF, to the performance of isolated **A1** and in situ generated (**A2**) [(SIMes)CuCF_3_] as well as to the [(PPh_3_)_3_CuCF_3_ + dtbpy] combination (**C1**) under their reported optimized solvent conditions to explore the effect of the solvent on the lower performing systems in Figure 1 [10,14]. The results are shown in Figure 2 and highlight the fact that the solvent plays a key role in reagent performance, even for reasonably comparable LCuCF_3_ complexes. The system **C1** shows high yields at early reaction times, but then suffers a severe leveling out effect after approximately ten hours. Based on the data, it is tempting to suggest that system **C1** might be worthy of a thorough mechanistic analysis, as if one can fully understand why the reagent suffers a rapid deactivation then a performance improvement may be possible. System **A1**, on the other hand, displays sluggish reactivity at early reaction times, but steadily produces 4-(trifluoromethyl)-1,1’-biphenyl in yields that are slightly higher than **C1** after 30 hours. [(SIMes)Cu(CF_3_)], generated in situ from [(SIMes)Cu(O*-t-*Bu)] and TMSCF_3_ (**A2**) afforded the highest yields at extended reaction times. It is interesting to note here, for the trifluoromethylation of 4-iodobiphenyl with SIMes copper complexes under the reported literature conditions, that the better performer was not the isolated and well-defined LCu–CF_3_ complex, but instead the LCu–CF_3_ complex generated in situ from the copper *tert*-butoxide precursor. This trend in reactivity mirrors that which was observed for the phen-based systems **B1** and **B2** in Figure 1. The reported optimized conditions for **A1** and **A2**, however, involve using copper as the limiting agent with a five-fold excess of aryl halide [4]. Therefore, while yields of the SIMes-based systems can provide good yields of product, the phen-based systems remain far more practical. Ligandless CuCF_3_ was also tested under the reported optimized reaction conditions [15], but in our hands the protocol afforded CHCF_3_ as the major fluorine-containing product.

**Figure 3:**
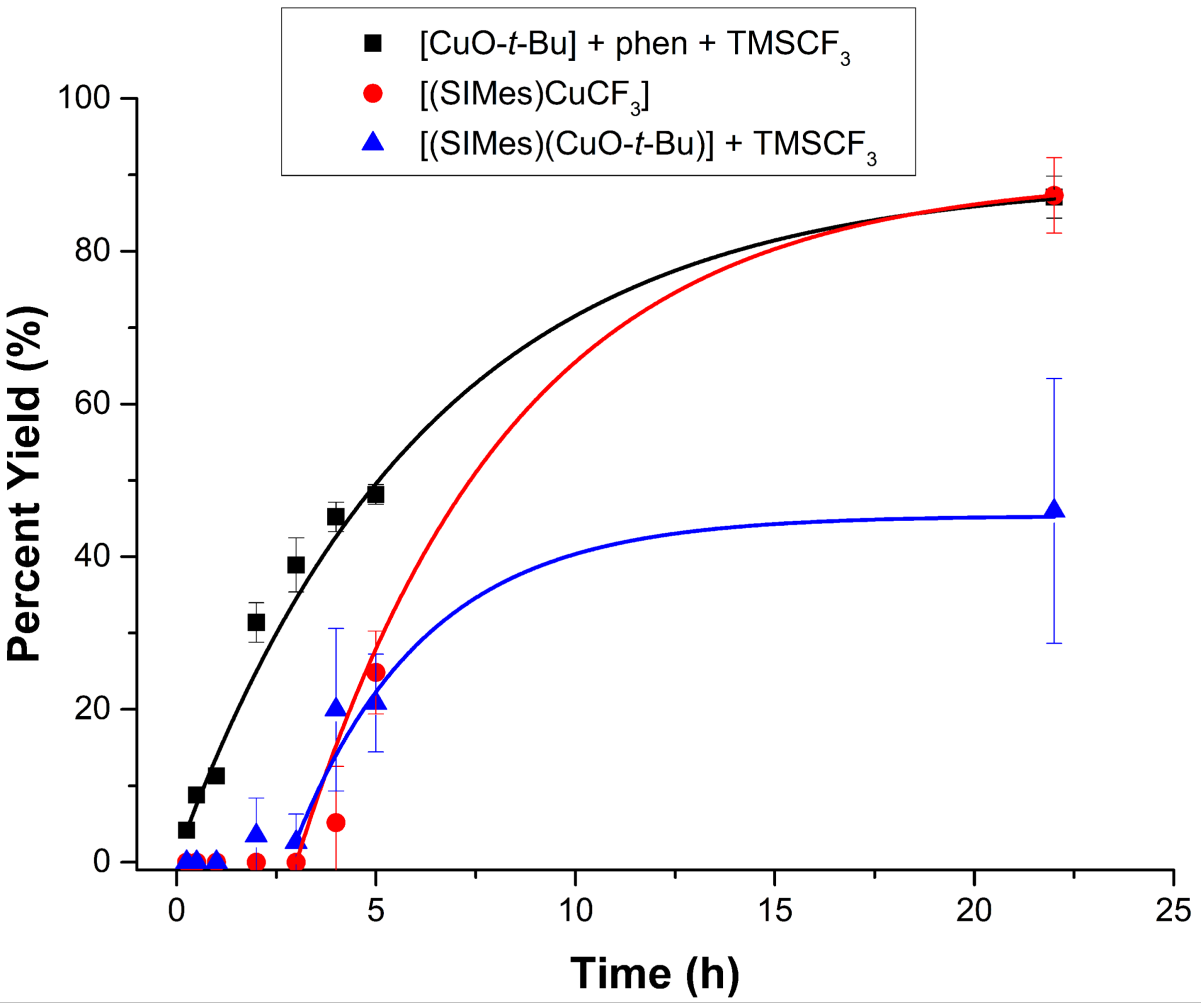
Reaction of 1-iodo-4-methylbenzene with systems **A1**, **A2**, and **B2** to produce 1-methyl-4-(trifluoromethyl)benzene. Conditions for **B2**: DMF, 50 °C. Conditions for **A1** and **A2**: DMI/benzene (1.5:7.5), 50 °C. Yields were calculated by ^19^F NMR spectroscopy versus an internal standard.

Because our group has developed the NHC-based copper reagents for trifluoromethylation reactions [10–11], we were interested in comparing the effects of electronics and sterics of the aryl halides using the NHC-based systems **A1** and **A2** with the phen system **B2**. First, in order to explore reactivities with more electron rich aryl iodides, we investigated the use of 4-iodotoluene as a substrate for trifluoromethylation reactions. Because the product 1-methyl-4-(trifluoromethyl)benzene had similar retention times as the solvents in the gas chromatography analyses, we monitored the reactions of the iodotoluenes by quantitative ^19^F NMR spectroscopy. Using the same solvent systems employed for the reactions in Figure 2, conversions were measured over a 22 hour period (Figure 3). In this case, [(SIMes)Cu(CF_3_)] performed just as well as the [(phen)Cu(CF_3_)] generated in situ at the 22 hour mark. However, the yield versus time plot revealed that the phen-based reagent was clearly better at early reaction times. The plots revealed other interesting information. For the electron-rich aryl iodides, the reactivity difference for the SIMes copper complexes was opposite from what was observed previously in Figure 2. Here, the isolated and well-defined [(SIMes)Cu(CF_3_)] outperformed the in situ-generated counterpart, although at the five hour mark both performed equally well. Single time point yields would not have been able to identify the leveling out of reactivity of **A2** for the more electron-rich aryl halide. Moreover, an induction period was observed for both **A1** and **A2**. We noted a detection limit of approximately 2% with our NMR spectrometer, so we believe this induction period with the electron-rich substrates is real and not stemming from the different analytical method used for determining yields for Figure 2 and Figure 3. Why an induction period is observed for the iodotoluenes but not for the iodobiphenyl substrate is still not well-understood and is currently under investigation.

**Figure 4:**
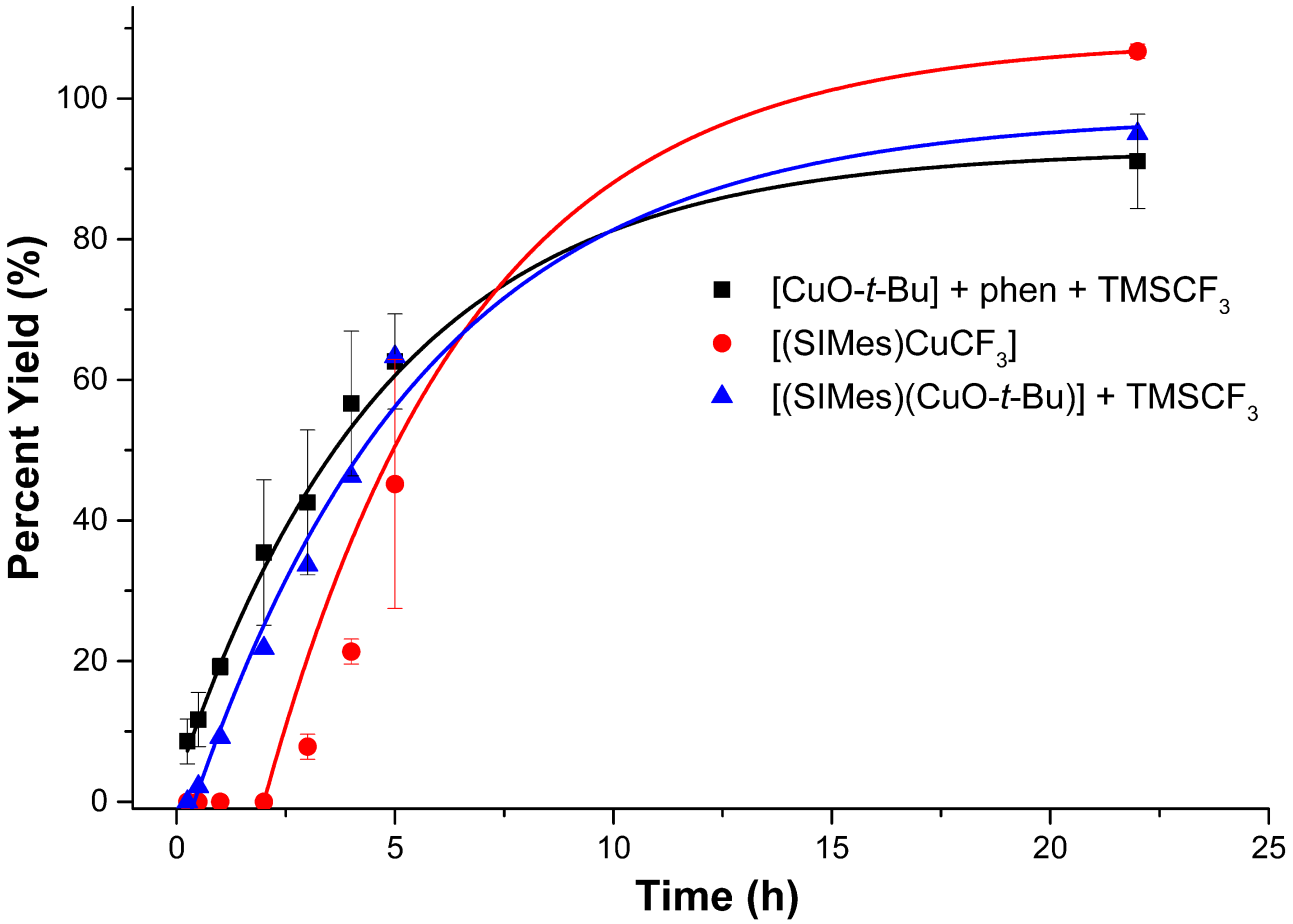
Reaction of 2-iodotoluene with systems **A1**, **A2**, and **B2** to produce 1-methyl-2-(trifluoromethyl)benzene. Conditions for **B2**: DMF, 50 °C. Conditions for **A1** and **A2**: DMI/benzene (1.5:7.5), 50 °C. Yields were calculated by ^19^F NMR spectroscopy versus an internal standard.

Reactions were then run with 2-iodotoluene in order to gauge steric effects in the trifluoromethylation reactions. It should be noted here that the promoting effect of *ortho* substituents in trifluoromethylation reactions is well-known. For example, the rate of trifluoromethylation of *o*-MeC_6_H_4_Br was found to be 3.5 times faster than that for bromobenzene by CuCF_3_ in DMF [16]. Figure 4 describes the results of the trifluoromethylations of 2-iodotoluene with systems **A1**, **A2**, and **B2**. For this iodoarene substrate, the phen- and the NHC-based copper reagents performed nearly equally well at almost all time periods. The well-defined and isolated **A1** again showed a noticeable induction period, whereas the induction period for **A2** was short.

## Conclusion

In summary, we have determined that in DMF the best trifluoromethylating agent was generated in situ using the [Cu(O*-t-*Bu)]_4_/Me_3_SiCF_3_/phen combination. However, when optimized solvents were employed, other metal complexes and precursors approached and even exceeded the [(phen)Cu(CF_3_)] system, albeit with much higher metal loadings. In order to rigorously assess future trifluoromethylating agents and minimize issues of reproducibility, we encourage others to provide comparative data (yields versus time) for any newly developed trifluoromethylation reaction with a well-established reagent using identical reaction vessels and performed by the same experimentalist. The work shown here reveals the importance of comparing trifluoromethylation reactions using a number of different variables (solvent, sterics, and electronics) in order to adequately describe a catalyst’s performance for the community. Explicit benchmarking in catalysis science is rarely reported in the literature (less than 500 mentions in approximately 1 × 10^6^ articles describing catalytic phenomena) [17], and as methodologies for trifluoromethylation reactions continue to develop it will be important to have protocols for assessing new reagents.

## Experimental

### General

[(Phen)Cu(CF_3_)] was purchased from Aspira Scientific (Lot #40C906, 90% purity) and used without further purification. All other copper reagents were prepared according to reported procedures and were verified by ^1^H NMR and ^19^F NMR for purity. Copper salt precursors were purchased from Sigma-Aldrich. Trimethyl(trifluoromethyl)silane (99% purity) was purchased from SynQuest Labs, Inc. and used without further purification. All other chemicals were verified by ^1^H NMR for purity and used without further purification. Purity of reagents used, CuCl (97%), KF (≥99%), 1,10-phenanthroline (≥99%), NaO-*t*-Bu (98%), KO-*t*-Bu (98%), K_2_CO_3_ (≥99%), undecane (≥99%), fluorobenzene (≥99%), 4-iodobiphenyl (97%), 4-iodotoluene (97%), 2-iodotoluene (97%), and 4,4′-di-*tert*-butyl-2,2′-dipyridyl (98%). All solvents were purified by passing through activated alumina and/or copper in a solvent purification system supplied by Pure Process Technology or purchased anhydrous from Fisher Scientific (toluene, acetonitrile, DMF, and DMI). The quantitative analyses were accomplished using a Shimadzu GC-2010 Plus Gas Chromatograph and flame ionization detector (FID). A Rxi-5ms (fused silica), low-polarity phase, crossbond diphenyl dimethyl polysiloxane, 15.0 m length column was used. Parameters were: injection volume of 4.0 µL, 25:1 split ratio, linear velocity of 57.0 cm/s, total flow of 65.3 mL/min, and temperature program starting at 40 °C held for one minute, followed by a temperature ramp of 20.0 °C per minute to the final temperature of 250 °C which was held for 4 minutes. All peaks were well separated. All manipulations were performed using standard Schlenk and high vacuum techniques or were performed in a nitrogen filled glovebox. The quantitative NMR analyses were accomplished using a Bruker Ascend 400 MHz spectrometer by ^19^F NMR spectra referenced to internal standard of fluorobenzene. Solution ^1^H NMR spectra were recorded at ambient temperature on a Bruker Ascend 400 MHz spectrometer and referenced to residual proton solvent signals. ^19^F NMR spectra were recorded on the Bruker Ascend NMR spectrometer operating at 376 MHz and referenced to trifluorotoluene set at δ −63.7. All graphical data were treated with a best fit curve generated by the Origin 9.0.0 program. The exponential fit with function ExpGro1 was selected for all data.

**Updated procedure for the preparation of [(SIMes)_2_Cu][Cu(CF_3_)_2_] (2):** A solution of [(SIMes)Cu(O-*t*-Bu)] (220 mg, 0.50 mmol) and CF_3_Si(CH_3_)_3_ (0.110 mL, 0.74 mmol) in 6.0 mL THF was stirred at room temperature. The conversion to product was monitored by ^19^F NMR spectroscopy, and after 1.5 h the volatiles were evaporated on a high vacuum line. The white residue was filtered and washed twice with 5 mL toluene and then twice with 5 mL of pentane. The yield of [(SIMes)_2_Cu][Cu(CF_3_)_2_] was 81%. The spectroscopic data matched literature values [10]. ^1^H NMR (25 °C, CD_2_Cl_2_) δ 1.84 (s, 12H), 2.39 (s, 6H), 3.80 (s, 4H), 6.89 (s, 4H); ^19^F NMR (25 °C, CD_2_Cl_2_) δ −31.33 (s, 3F).

**Updated procedure for the preparation of [(SIMes)Cu(O*-t-*Bu)] (3):** A suspension of [(SIMes)CuCl] (330 mg, 0.81 mmol) and *t*-BuONa (78 mg, 0.81 mmol) in 6.0 mL THF was stirred for 2 h at room temperature and then filtered through a pad of Celite. The Celite was washed two times with 4 mL of THF. The solvents were then removed on a high vacuum line, and the resulting light yellow residue was dissolved in benzene and then filtered again through a pad of Celite. The Celite was washed two times with 4 mL of benzene, and the filtrate was evaporated on a high vacuum line. The resulting white solid was washed with pentane, filtered, and dried. Yield 92%. The spectroscopic data matched literature values [10]. ^1^H NMR (C_6_D_6_) δ 1.31 (s, 9H), 2.12 (s, 6H), 2.14 (s, 12H), 3.01 (s, 4H), 6.73 (s, 4H).

**General procedure for the standard conditions of trifluoromethylation of 4-iodobiphenyl (systems A1, B1, and C1 in** Figure 1**).** To a 20 mL vial was added copper trifluoromethyl reagent (0.28 mmol) in 5.4 mL of DMF. In the case of [(PPh_3_)_3_CuCF_3_] [14], 77.2 mg (0.28 mmol) of dtbpy was also added. Then 67.1 mg (0.23 mmol) of 4-iodobiphenyl and 60.5 µL (0.28 mmol) of undecane as internal standard, were added to the vial. The solution was then allowed to stir for five minutes. Then 0.6 mL aliquots were taken and transferred into 5 mL air-tight ampules fitted with a stirring bar. The ampules were sealed and placed in an oil bath at 50 °C. The reactions were removed from the oil bath at various time intervals and quenched with 0.6 mL of methanol in air. Aliquots of each solution were injected into a GC-FID and the reactions were monitored for the formation of the 4-trifluoromethylbiphenyl product.

**General procedure for the standard conditions of trifluoromethylation of 4-iodobiphenyl using in situ generated reagents (systems B2, B3, and D1 in** Figure 1**).** The preparation of each reagent (**B2**, **B3**, and **D1**) is described below. After preparation of the reagent, 60.50 µL (0.2851 mmol) of undecane was added as internal standard. Each solution was allowed to stir for five minutes, and then 0.60 mL aliquots were taken and transferred into 5 mL air-tight resealable ampules. The ampules were sealed and placed in an oil bath at 50 °C and the solutions were stirred. The reactions were removed from the oil bath at various time intervals and quenched with 0.6 mL of methanol in air. Aliquots of each solution were injected into a GC-FID and the reactions were monitored for the formation of the 4-trifluoromethylbiphenyl product.

**Generation of B2 in** Figure 1**:** For the generation of (phen)CuCF_3_ in situ, a vial was charged with 28.4 mg (0.28 mmol) of CuCl, 32.3 mg (0.28 mmol) of KO-*t-*Bu, and 51.5 mg (0.28 mmol) of 1,10-phenanthroline in 5.4 mL of DMF. The solution was stirred for 0.5 h before the addition of 0.042 mL (0.28 mmol) of (Me)_3_SiCF_3_. The solution was stirred for an additional hour before the introduction of 0.23 mmol of 4-iodobiphenyl.

**Generation of B3 in** Figure 1**:** A vial was charged with 0.232 mmol of 4-iodobiphenyl, 53.6 mg (0.28 mmol) of CuI, 60.5 mg (0.28 mmol) of [K][B(OMe_3_)(CF_3_)] [8], and 51.5 mg (0.28 mmol) of 1,10-phenanthroline in 5.4 mL of DMF.

**Generation of D1 in** Figure 1**:** A vial was charged with (0.23 mmol) of 4-iodobiphenyl, 53.6 mg (0.28 mmol) of CuI, 16.7 mg (0.28 mmol) of KF, and 0.042 mL (0.28 mmol) of (Me)_3_SiCF_3_ in 5.4 mL of DMF as solvent.

### General procedure for the ‘best’ conditions of trifluoromethylation of 4-iodobiphenyl (systems **A1**, **A2**, and **C1** in Figure 2)

**Reaction conditions employing [(SIMes)_2_Cu][Cu(CF_3_)_2_] (system A1):** A vial was charged with 1.15 mmol of 4-iodobiphenyl and 105 mg (0.12 mmol) of [(SIMes)_2_Cu][Cu(CF_3_)_2_] in 5.4 mL of DMI/benzene (1.5:7.5) with 60.5 µL (0.29 mmol) of undecane, as internal standard. After the solution was allowed to stir for five minutes, 0.6 mL aliquots were taken and transferred into 5 mL air-tight ampules. The ampules were sealed and placed in an oil bath at 50 °C. The reactions were removed from the oil bath at various time intervals and quenched with 0.6 mL of methanol in air. Aliquots of each solution were injected into a GC-FID and the reactions were monitored for the formation of the 4-trifluoromethylbiphenyl product.

**Reaction conditions employing [(SIMes)Cu(O*-t-*Bu)] + TMSCF_3 _(system A2):** A vial was charged with 1.15 mmol of 4-iodobiphenyl, 106 mg (0.24 mmol) of [(SIMes)Cu(O*-t-*Bu)] and 0.053 mL (0.359 mmol) of (Me)_3_SiCF_3_ in 5.4 mL of DMI/benzene (1.5:7.5) with 60.5 µL (0.29 mmol) of undecane, as internal standard. After the solution was allowed to stir for five minutes, 0.6 mL aliquots were taken and transferred into 5 mL air-tight ampules. The ampules were sealed and placed in an oil bath at 50 °C. The reactions were removed from the oil bath at various time intervals and quenched with 0.6 mL of methanol in air. Aliquots of each solution were injected into a GC-FID and the reactions were monitored for the formation of the 4-trifluoromethylbiphenyl product.

**Reaction conditions employing [(PPh_3_)_3_CuCF_3_] (system C1):** A vial was charged with 0.31 mmol of 4-iodobiphenyl, 264 mg (0.29 mmol) of (PPh_3_)_3_CuCF_3_, and 85.0 mg (0.32 mmol) of dtbpy in 5.4 mL of toluene with 60.5 µL (0.29 mmol) of undecane, as internal standard. After the solution was allowed to stir for five minutes, 0.6 mL aliquots were taken and transferred into 5 mL air-tight ampules. The ampules were sealed and placed in an oil bath at 80 °C. The reactions were removed from the oil bath at various time intervals and quenched with 0.6 mL of methanol in air. Aliquots of each solution were injected into a GC-FID and the reactions were monitored for the formation of the 4-trifluoromethylbiphenyl product.

### General procedure for the trifluoromethylation of 2-iodotoluene and 4-iodotoluene (system **A1**, **A2** and **B2** in Figure 3 and Figure 4)

**Reaction conditions employing [(SIMes)_2_Cu][Cu(CF_3_)_2_] (system A1):** A vial was charged with 270 mg (1.20 mmol) of 2-iodotoluene or 4-iodotoluene and 105 mg (0.12 mmol) of [(SIMes)_2_Cu][Cu(CF_3_)_2_] in 5.4 mL of DMI/benzene (1.5:7.5) with 90.0 µL (0.95 mmol) of fluorobenzene, as internal standard. After the solution was allowed to stir for five minutes, 0.6 mL aliquots were taken and transferred into 5 mL air-tight ampules. The ampules were sealed and placed in an oil bath at 50 °C. The reactions were removed from the oil bath at various time intervals and quenched with 0.6 mL of methanol in air. Each aliquot was monitored by ^19^F NMR spectroscopy for formation of the respective 2-trifluoromethyltoluene or 4-trifluoromethyltoluene product.

**Reaction conditions employing [(SIMes)Cu(O-*t-*Bu)] + TMSCF_3_ (system A2):** A vial was charged with 270 mg (1.20 mmol) of 2-iodotoluene or 4-iodotoluene, 106 mg (0.24 mmol) of [(SIMes)Cu(O*-t-*Bu)] and 0.053 mL (0.36 mmol) of (Me)_3_SiCF_3_ in 5.4 mL of DMI/benzene (1.5:7.5) with 90.0 µL (0.95 mmol) of fluorobenzene, as internal standard. After the solution was allowed to stir for five minutes, 0.60 mL aliquots were taken and transferred into 5 mL air-tight ampules. The ampules were sealed and placed in an oil bath at 50 °C. The reactions were removed from the oil bath at various time intervals and quenched with 0.6 mL of methanol in air. Each aliquot was monitored by ^19^F NMR spectroscopy for formation of the respective 2-trifluoromethyltoluene or 4-trifluoromethyltoluene product.

**Reaction conditions employing [(phen)Cu(O-*t*-Bu)]_4_ + TMSCF_3_ (system B2):** For the generation of (phen)CuCF_3_ in situ, a vial was charged with 28.4 mg (0.28 mmol) of CuCl, 32.3 mg (0.28 mmol) of KO-*t-*Bu, and 51.5 mg (0.28 mmol) of 1,10-phenanthroline. To the vial, 5.4 mL of DMF was added. The solution was stirred for 0.5 h before the addition of 0.042 mL (0.28 mmol) of Me_3_SiCF_3_. The solution was stirred for an additional hour before the introduction of 52.1 mg (0.23 mmol) of 2-iodotoluene or 4-iodotoluene and 90.0 µL (0.95 mmol) of fluorobenzene, as internal standard. After the solution was allowed to stir for five minutes, 0.60 mL aliquots were taken and transferred into 5 mL air-tight ampules. The ampules were sealed and placed in an oil bath at 50 °C. The reactions were removed from the oil bath at various time intervals and quenched with 0.6 mL of methanol in air. Each aliquot was monitored by ^19^F NMR spectroscopy for formation of the respective 2-trifluoromethyltoluene or 4-trifluoromethyltoluene product.
